# Evaluating rural health outcomes: A methodological approach using population‐level data

**DOI:** 10.1002/hcs2.94

**Published:** 2024-05-07

**Authors:** Gal Av‐Gay, Anshu Parajulee, Kathrin Stoll, Jude Kornelsen

**Affiliations:** ^1^ Department of Family Practice, Faculty of Medicine University of British Columbia Vancouver British Columbia Canada

**Keywords:** evaluation methodology, administrative data uses, epidemiology, cohort analysis

## Abstract

**Background:**

The sustainability of rural surgical and obstetrical facilities depends on their efficacy and quality of care, which are difficult to measure in a rural context. In an evaluation of rural practice, it is often the case that the only comparators are larger referral facilities, for which facility‐level comparisons are difficult due to differences in population demographics, acuity of patients, and services offered. This publication outlines these limitations and highlights a best‐practice approach to making facility‐level comparisons using population‐level data, risk stratification, tests of noninferiority, and Firth logistic regression analysis. This includes an investigation of minimum sample‐size requirements through Monte Carlo power analysis in the context of low‐acuity rural surgical care.

**Methods:**

Monte Carlo power analysis was used to estimate the minimum sample size required to achieve a power of 0.8 for both logistic regression and Firth logistic regression models that compare the proportion of surgical adverse events against facility type, among other confounders. We provide guidelines for the implementation of a recommended methodology that uses risk stratification, Firth penalized logistic regression, and tests of noninferiority.

**Results:**

We illustrate limitations in facility‐level comparison of surgical quality among patients undergoing one of four index procedures including hernia repair, colonoscopy, appendectomy, and cesarean delivery. We identified minimum sample sizes for comparison of each index procedure that fluctuate depending on the level of risk stratification used.

**Conclusion:**

The availability of administrative data can provide an adequate sample size to allow for facility‐level comparisons in surgical quality, at the rural level and elsewhere. When they are made appropriately, these comparisons can be used to evaluate the efficacy of general practitioners and nurse practitioners in performing low‐acuity procedures.

## Graphical abstract

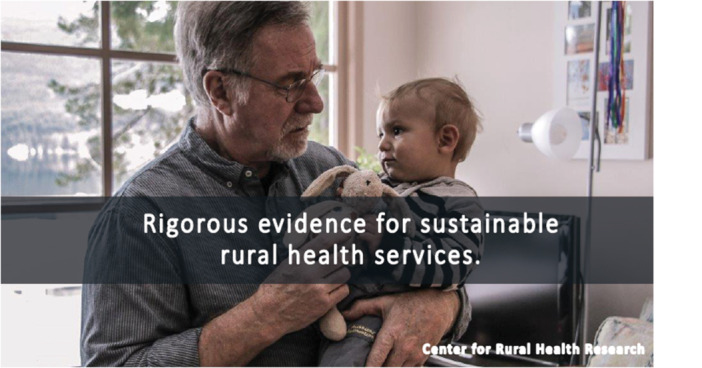

**Abbreviations**
CCI: Canadian Classification of Health Interventions code
CIHI: Canadian Institute for Health Information
CMG: CIHI Case Mix Group
HHF: Hospital Harms Framework
ICD‐10‐CA: International Statistical Classification of Diseases and Related Health Problems, Tenth Revision, Canada ICD‐10‐CA
PSBC: Perinatal Services BC
RSON: British Columbia's Rural Surgical and Obstetrical Network

## 1 BACKGROUND

The evaluation of British Columbia's Rural Surgical and Obstetrical Networks (RSON) [1] aims to investigate whether the quality of care at British Columbia (BC)'s rural surgical sites is comparable to that of larger referral facilities, specifically for low‐acuity, high‐volume procedures. In addition to experiencing lower volumes and treating lower risk patients compared to the larger referral facilities, these rural sites largely rely on visiting specialists, family physicians with enhanced surgical skills, and nonphysician clinicians such as nurse practitioners. These and other factors need to be considered to appropriately make facility‐level comparisons, so as to better inform healthcare policy and decision‐making.

We propose a methodology that uses population‐level data made available by Population Data BC [2] via the Canadian Institute for Health Information [3] and Perinatal Services BC [4]. To assess the facility‐level quality of care, adverse events are compared for patients receiving one of four index procedures: hernia repair, colonoscopy, appendectomy, and cesarean delivery. We describe issues inherent in an evaluation of this nature and outline a methodology that accounts for these issues, which include the choice and mapping of adverse events associated with each index procedure, limitations of rare‐event analysis, controlling for bias in facility‐level comparisons, and appropriate statistical tests.

### 1.1 Choice of primary outcomes

The selection of relevant adverse events for each index procedure is an epidemiological question that requires clinician consultation; however, these selections are ultimately limited by data availability and coding practice at the facility level. Using a Delphi survey [5], adverse events were selected and then mapped to ICD‐10‐CA (International Statistical Classification of Diseases and Related Health Problems, Tenth Revision, Canada ICD‐10‐CA) and CCI (Canadian Classification of Health Interventions code) codes. This mapping was informed by previous literature concerning the use of ICD‐10‐CA and CCI codes to identify surgical complications [6, 7, 8]. Volumes of both the procedures and their respective adverse events were tabulated and presented for spot‐checking by clinicians at the study sites, after which changes were made to the coding based on the accuracy compared with clinician records.

### 1.2 Sample size requirements for analysis of rare events

When comparing the proportion of adverse events between rural and referral facilities, there are two important concerns affecting minimum sample size requirements. First, adverse surgical events are quite rare, meaning that hypothesis tests will be very limited in their power even at the relatively high sample sizes found in population‐level data. Second, the data will likely be unbalanced, with referral facilities having a much higher number of cases than rural facilities, meaning that comparisons that control for additional explanatory variables will be prone to “separation” or “quasiseparation” [9].

To achieve an appropriate sample size and to avoid separation, our methodology combines all adverse events to obtain an aggregate “adverse event proportion” for each index procedure. The issue with combining adverse events is that the relative severity of each event is not accounted for, and for this reason, we highly recommend including the raw proportion of each individual adverse event in addition to any combined analysis results. Using the combined adverse event proportions as the primary outcome, we include results of a Monte Carlo simulation power analysis [10] to describe sample size requirements for each index procedure, as well as power limitations for the available data. Furthermore, we propose the use of “Firth” penalized logistic regression [11] and show its improved power in comparison with logistic regression, specifically for small sample sizes.

### 1.3 Hospital selection bias

Making appropriate comparisons requires consideration of confounders, as higher acuity patients often travel to a referral site over their local rural facility. Age, sex, socioeconomic status, and the health status of a patient can play an important role in determining what facility a patient will choose to receive care at. The health status of a patient can be measured using risk stratification [12], for which there are a number of tools available that provide some capacity to control for the differences in the acuity of cases between rural and referral facilities. These tools include CIHI's case mix group comorbidity levels [13], weighted antepartum risk scores for perinatal cases [14], and the Charlson comorbidity index [15], among others.

Inclusion of these risk categories as a confounding factor in a regression model poses a conceptual issue, in that the individual adverse events that comprise the primary outcome are often used to define the risk groups themselves, resulting in artificially inflated correlation between the response (adverse event) and confounder (risk category). We therefore recommend stratifying the analysis by these risk groups by conducting multiple parallel analyses for different subsets of the population based on their risk category. It is important to present the individual adverse event proportions for the different risk groups to illustrate how stratification is affecting the volume of each adverse event. This stratified analysis also needs to be considered in sample size considerations, as the proportion of adverse events will be lower in the low‐risk groups.

### 1.4 Issues with conventional hypothesis testing

The results of logistic regression analyses, including the “Firth” penalized logistic regression models, are conventionally presented as adjusted odds ratios, confidence intervals, and *p* values. For the RSON evaluation, these adjusted odds ratios describe the odds of experiencing an adverse event at a rural facility compared to a referral facility, adjusted for confounding factors. A low *p* value conventionally provides evidence against the null hypothesis: that the odds of experiencing an adverse event are equivalent at rural and referral facilities. This does not mean that a higher *p* value provides evidence in favor of the null, but rather that we have merely failed to disprove it. Therefore, to ask whether patients have similar odds of experiencing adverse events at rural and referral facilities, we are required to rephrase the null hypothesis in terms of tests of noninferiority/equivalence [16] instead of those for tests of inferiority/superiority. We describe a best‐practice approach to the implementation of noninferiority testing commonly used in clinical trials, including the choice of noninferiority margin and application to logistic regression adjusted odds ratios and raw adverse‐event proportions.

## 2 METHODS

Four index procedures were used to evaluate the quality of care at rural facilities, selected based on their relatively high rural volumes and low acuity. These procedures include colonoscopy, appendectomy, hernia repair, and cesarean delivery. Through clinician engagement, a set of system‐level adverse events were identified that were associated with each intervention (see Table 1). These events were then mapped to relevant ICD‐10‐CA and CCI codes based on similar approaches in the literature [6, 7, 8]. The proportions of adverse events for each facility were derived using an initial mapping, which was revised after consultation with rural clinicians in the RSON sites regarding the accuracy of the rates.

**Table 1:** List of adverse events for each procedure.

Intervention specific events	General and anesthesia events
**Appendectomy** Gastrointestinal complication **Colonoscopy** Heavy bleeding postprocedure	**Anesthesia events** Aspiration pneumonia Failed tracheal intubation Medication error, wrong dose Problems with airways in postanesthesia recovery, excluding aspiration pneumonia
**C‐section** Bladder, urethra injury Puerperal sepsis, including endometritis Wound disruption **Hernia repair** Hematoma and hemorrhage **Neonatal** Birth trauma	**General events** Acute renal failure Bladder injury Blood transfusion Bowel injury Cerebrovascular accident (stroke) Cut, puncture, perforation, or hemorrhage Deep vein thrombosis Foreign body Myocardial infarction Pulmonary embolism Sepsis Surgical site infection Wound disruption

*Note*: General and anesthesia adverse events were included for all procedures.

### 2.1 Delphi process

We engaged rural clinicians and other provincial key stakeholders with expertise in quality improvement in the selection of adverse event indicators to measure the quality of surgical care. To generate multidisciplinary expert consensus on quality measures for rural general surgery in BC, we implemented a two‐round modified Delphi process [5].

The indicators we included broadly aligned with those listed in the Canadian Hospital Harms Framework (HHF) [17]. It should be noted that many of the HHF indicators were not relevant due to a number of factors including the short duration of hospital stay at RSON rural facilities, making it less likely for patients to be exposed to hospital harms.

### 2.2 Data mapping

We requested population‐level hospital data from Population Data BC [2] through CIHI—Canadian Institute for Health Information [3]. These data contained all hospital visits at rural and referral RSON facilities between January 1, 2016 and March 31, 2021; accessed between August 3, 2022 and April 1, 2023. Each record contained up to 25 diagnosis codes (ICD‐10‐CA), 20 intervention codes (Canadian Classification of Health Interventions code), as well as codes for patient transfer, provider specialty, admission to the operating room (OR) and emergency room (ER), and a discrete measure of patient comorbidity level using CIHI Case Mix Group (CMG) [13] methodology. Patient adverse events were identified using all patient hospital visits within 30 days of the initial procedure.

To identify index procedures and outcomes of interest among the data, we determined which CCI codes and ICD‐10 codes corresponded to each index procedure and their corresponding health outcomes. To conduct this “mapping,” we referred to codes used by other groups including those that appeared in peer‐reviewed surgical outcome literature [6, 7, 18]. Where we could not find codes published by other groups, we used relevant search terms within the CCI or ICD‐10 codebooks and consulted with rural clinicians about the appropriateness of these codes. When databases included non‐ICD fields for outcomes (e.g., death), we chose to use these fields instead of ICD‐10 codes. For some outcomes such as unplanned intubation and deep surgical site infection, we were not able to identify corresponding codes/fields in the databases. Please refer to Table S1 for a list of the codes that we used.

### 2.3 Clinician engagement

We consulted with clinicians after the Delphi and mapping processes. This involved obtaining feedback from rural clinicians at RSON hospitals on the face validity of preliminary findings. For instance, one RSON hospital communicated that the rate of postcolonoscopy heavy bleeding that we reported for their site was much higher than what they remembered for the data timeframe. Review of colonoscopy outcome literature from Canada and other high‐income countries also indicated that the rates for heavy bleeding across the hospitals in our study were unusually high. These inconsistencies prompted us to re‐examine the appropriateness of the three hemorrhage codes we were using for this outcome. We decided to only keep the hemorrhage code that was specifically a postprocedure complication (T810) and removed the other two general hemorrhage codes (K625 and K922).

### 2.4 Analysis of rare events

Adverse events in Table 1 are rare, which can lead to difficulty when modeling using conventional approaches (e.g., logistic regression) due to issues of (quasi) separation leading to degenerate effect estimates [9]. We therefore use combined measures for adverse events, where for each patient we construct an indicator for whether any adverse events occurred. The limitation of this combined adverse event indicator is that the relative severity of individual adverse events is not accounted for. For this reason, we highlight the importance of presenting the individual adverse event proportions for each facility type, allowing for a more transparent understanding of what is being compared between facility types. As even the combined adverse event indicator is still quite rare, we recommend using Firth penalized logistic regression [11], which has been shown to have reduced bias in modeling rare events where there is possible separation [9]. This is further illustrated in the power analysis included below.

### 2.5 Controlling for bias

When comparing surgical outcomes from rural facilities to those at larger referral facilities, we must address the potential for bias, as higher acuity cases are often referred to larger referral hospitals that have a higher tier of service, resulting in a higher number of expected adverse events at referral facilities. This can be compounded by patient selection bias, where high‐risk patients may choose to receive care at a larger referral hospital over their local rural facility. Age, sex, socioeconomic status, as well as procedure‐specific attributes can be included as explanatory variables in logistic regression analyses to account for their possible confounding effect on the relationship between facility type and patient adverse events. However, these factors may not fully explain differences in patients between facilities. To help quantify the amount of unmeasured confounding that would be needed to explain away our effects, we recommend *E* values [19]. An *E* value tells us the minimum strength an unmeasured confounder needs to have with the outcome and treatment to explain away the observed effect of the treatment variable. We recommend presenting *E* values for the logistic regression adjusted odds ratios, corresponding to both their point estimate as well as the upper limit of the 95% confidence interval. A higher *E* value tells us that a stronger unmeasured confounding effect will be required to explain away the effect of the treatment variable. Higher *E* values should therefore be interpreted as providing more evidence against the null hypothesis, or in favor of the relationship described by the adjusted odds ratio.

There are a number of other tools available that measure the health status of a patient, including the Charlson comorbidity index [15] and CIHI's Case Mix Group Comorbidity Levels [13]. As the coding of these tools utilizes ICD and CCI‐coded adverse events, including some defined in Table 1, it would not make conceptual sense to include them as predictors in a regression analysis where the response is the incidence of an adverse event. This can lead to inflated correlation between the response (incidence of an adverse event) and the confounder (health status), and will likely diminish the strength of other effects included in the model. Nonetheless, these measures of health status can still be leveraged through their use in risk stratification [12] of the study population into several strata according to their levels of patient comorbidity/risk. Care should be taken to illustrate how individual adverse events are distributed across the risk strata. Additionally, any sample size and power considerations must take these risk strata into account, as the proportion of adverse events will likely be lower among the lower risk strata.

We therefore use risk stratification to control for differences in patient health status, in addition to the inclusion of other confounders in logistic regression models, allowing us to compare the outcomes of patients between groups (e.g., RSON and referral facilities) for various risk strata. Using this approach, we estimate the odds that a patient will experience an adverse event given their risk strata and other confounding factors, calculated as an adjusted odds ratio. We use the CIHI Case Mix Group (CMG) Comorbidity Levels to stratify the study population into five risk strata, where the highest‐risk strata include all available patient data, and lower risk strata exclude certain high‐comorbidity patients. CMG comorbidity level (CL) ranges from 0 to 4 and is based on the expected cost impact of certain comorbidities on patient stay (length of stay and/or treatment). These comorbidities are derived from a diagnosis identified by ICD‐10‐diagnosis codes other than the most responsible diagnosis for a patient's hospitalization. Only patients with diagnosed conditions outside of the most‐responsible diagnosis that significantly increase resource requirements will have a CL > 0. Higher CL levels correspond to a greater risk for complication, and patients with a CL of 1 or higher, are expected to have at least 25% higher costs as a result of one or more comorbidities compared to costs for a similar patient without comorbidities.

## 3 RESULTS

We implemented a Monte Carlo simulation‐based power analysis to compare the sample size requirements using both Firth penalized regression and logistic regression, as well as to estimate the power of our analyses given the available data. Patient visits were simulated separately for rural and referral facilities based on their existing distributions from the population‐level data made available by Population Data BC [2], stratified by CIHI patient comorbidity level [13]. This power analysis assumes that the observed differences in raw proportions of adverse events between rural and referral facilities are in fact true and then proceeds to ask the questions of (1) how much power can be achieved using the available data and (2) how many samples (approximately) are required to achieve a power of 0.8, given the rarity of adverse events, the lack of balance in number of samples across the exposures (rural vs. referral), and the existing distributions of other confounding factors included in the model.

For each index procedure, the following steps were taken:

a. The data were separated into the four risk‐stratified groups based on the patient comorbidity level, with the first group containing only patients having a comorbidity level of 0, the second group containing patients having a comorbidity level of 0 and 1, and so on until the fifth group which contained all comorbidity levels (0–4).
b. For each risk strata, we calculated the proportion of rural patient visits, the proportion of adverse events, the proportion of women, as well as the means and standard deviations for age and income decile for both rural and referral facilities. For hernia repair and appendectomy, the proportion of laparoscopies and for cesarean birth gravidity and emergency versus elective cesarean birth were also obtained.
c. For each risk strata, data from all variables of sizes ranging from 1000 to 100,000 were simulated independently from the normal and binomial distributions based on their characteristics obtained in the previous step, for rural and referral facilities separately and in an amount proportional to their relative volumes. For each sample size, 1000 replications were simulated, and for each replication, both a logistic regression and a Firth logistic regression were fit. The proportion of significant *p* values (<0.05) from these 1000 replications was used to determine the power of the test for the given sample size. In the case of Firth logistic regression, a *p* value was obtained using a likelihood ratio test on the adjusted odds ratio, whereas for logistic regression, a *p* value is obtained using a Wald test on the adjusted odds ratio.
d. Using separate 1000 replications, we estimate the power for both logistic regression analysis and Firth logistic regression analysis at the available sample size, with data simulated as per instructions in the previous step.

We can see from Table 2 that Firth penalized logistic regression can achieve higher power than logistic regression analysis. This difference is most prominent for smaller sample sizes.

**Table 2:** Results of power analysis for all four index procedures, including minimum sample size required for power of 0.8, as well as simulated power estimate for available data for each risk strata.

Total available data	CMG comorbidity levels included	Rural proportion of adverse events	Referral proportion of adverse events	Sample size required for power of 0.8 using logistic regression	Sample size required for power of 0.8 using Firth penalized logistic regression	Logistic regression power estimate for available *n*	Firth logistic regression power estimate for available *n*
Hernia repair							
7460 (994 rural: 13.3%)	0	0.012	0.017	35,620	35,620	0.144	0.165
7634 (998 rural: 13.1%)	0,1	0.012	0.024	9000	8340	0.686	0.709
7706 (999 rural: 13.0%)	0,1,2	0.012	0.029	4550	4120	0.938	0.947
7753 (999 rural: 12.9%)	0,1,2,3	0.012	0.034	3340	3110	0.992	0.994
7792 (999 rural: 12.8%)	0,1,2,3,4	0.012	0.038	2810	2810	1.000	1.000
Appendectomy							
2392 (134 rural: 5.6%)	0	0.030	0.032	>100,000	>100,000	0.025	0.046
2534 (135 rural: 5.3%)	0,1	0.037	0.063	10,350	10,240	0.146	0.218
2587 (135 rural: 5.2%)	0,1,2	0.037	0.075	6530	6530	0.295	0.381
2607 (136 rural: 5.2%)	0,1,2,3	0.044	0.081	6720	6720	0.264	0.330
2613 (136 rural: 5.2%)	0,1,2,3,4	0.044	0.083	6510	6510	0.309	0.371
Colonoscopy							
51,319 (10,513 rural: 20.5%)	0	0.004	0.005	>100,000	>100,000	0.307	0.312
51,601 (10,535 rural: 20.4%)	0,1	0.005	0.007	40,020	40,020	0.870	0.874
51,780 (10,550 rural: 20.4%)	0,1,2	0.005	0.009	18,540	18,420	0.994	0.995
51,891 (10,558 rural: 20.3%)	0,1,2,3	0.005	0.010	11,180	11,020	1.000	1.000
51,968 (10,559 rural: 20.3%)	0,1,2,3,4	0.005	0.012	8180	8.070	1.000	1.000
Cesarean delivery							
5183 (852 rural: 16.4%)	0	0.012	0.015	>100,000	>100,000	0.094	0.109
5801 (928 rural: 16.0%)	0,1	0.016	0.020	>100,000	>100,000	0.084	0.093
5953 (937 rural: 15.7%)	0,1,2	0.018	0.021	>100,000	>100,000	0.054	0.058
5962 (937 rural: 15.7%)	0,1,2,3	0.018	0.021	>100,000	>100,000	0.079	0.088
5972 (937 rural: 15.7%)	0,1,2,3,4	0.018	0.021	>100,000	>100,000	0.067	0.076

Abbreviation: CMG, CIHI Case Mix Group.

## 4 DISCUSSION

Collecting, understanding, and documenting patient outcomes of health service delivery models is an essential part of ensuring safety and quality care across all health service domains. This becomes difficult when adverse events are compared across different service delivery levels in health systems where tiers of service are designed to correspond to increasing clinical need based on patient and condition complexity. Through the practice of accounting for patient and procedure confounders, in conjunction with a risk‐stratified analysis, much of this expected variance can be controlled for, allowing for a more robust comparison. Two fundamental issues remain, those being the limitations resulting from rare adverse events, and the lack of clinical relevance of traditional hypothesis testing. When designing and implementing services, the salient question is one of equivalence: can patients expect to receive the same quality of care for equivalent procedures regardless of where they receive the care and who provides it?

### 4.1 Issues with traditional hypothesis testing

A low *p* value (e.g., <0.05) associated with adjusted odds ratios provides evidence against the null hypothesis—that there is no difference in the odds of experiencing an adverse outcome between study groups. In an evaluation of rural facilities compared to larger referral facilities, it is not necessarily desirable to provide evidence against this null hypothesis, showing that patients at rural facilities exhibit significantly lower or higher odds of experiencing an adverse event than those at higher tiered facilities. The intention is rather to show that rural facilities are at least as good as referral facilities, after controlling for differences in comorbidity level and other confounders.

The issue is in interpreting a nonsignificant *p* value from a test of superiority/inferiority: consider a situation where we observe that the rate of bladder injury from appendectomy was higher at rural facilities than at referral facilities. Using a conventional hypothesis test, we investigate whether this difference is significant and find a high *p* value, therefore failing to reject the null hypothesis. It would be incorrect to infer from this high *p* value that patients at rural facilities exhibit odds of an adverse event that are not worse than that of patients from referral facilities, as a high *p* value does not provide evidence in favor of the null hypothesis. We must instead use a test of noninferiority, for which the null hypothesis asks whether the odds of an adverse event at rural facilities are not any higher than some predetermined “noninferiority” margin.

Tests of noninferiority require specification of a “noninferiority” margin, which corresponds to some threshold on the proportion of adverse events/odds of adverse events in the control group [20]. The noninferiority margin is essentially an allowable difference in our outcome of interest between the treatment group and the active control, such that if the observed difference is within this margin, we have evidence in favor of noninferiority of the treatment. We assign the noninferiority margin using the 95% confidence interval for the difference between the control and treatment groups [16] and implement noninferiority testing on our logistic regression/Firth penalized logistic regression coefficients.

### 4.2 Tests of noninferiority

Conventionally, a hypothesis test is used to evaluate the superiority of one treatment (e.g., rural facilities) compared to a control (referral facilities). Consider the case where *τ*_t_ is the treatment group proportion (e.g., proportion of adverse events at rural sites) and *τ*_c_ is the control group proportion (e.g., proportion of adverse events at referral sites). These tests often take on the form:

(1) $$\begin{array}{l} H_{0}:\tau_{t}-\tau_{c}=0,\\ H_{1}:\tau_{t}-\tau_{c}\neq 0. \end{array}$$

A common misconception is that a nonsignificant *p* value from a test (1) can be used as evidence in support of the lack of difference (equivalence) between the treatment and control. For example, it would be incorrect to interpret a nonsignificant *p* value as evidence in favor of the equivalence of the rates of adverse events between rural and referral facilities. Greene et al. [21] evaluated 88 reports of distinct studies from the literature published between 1992 and 1996 that investigated or concluded equivalence. They found that for 67% (59 of 88) of the studies, equivalence was claimed after a test for superiority yielded a nonsignificant result. Only 23% of the reports that were analyzed used an appropriate statistical test with predefined “equivalence boundary” or noninferiority margin to declare equivalence.

As we are interested in showing that outcomes at RSON sites are not worse, or “noninferior” to the control, a test of noninferiority is optimal. Tests of noninferiority test hypotheses such as the following:

(2) $$\begin{array}{l} H_{0}:\tau_{t}-\tau_{c}\geq \delta ,\\ H_{1}:\tau_{t}-\tau_{c}<\delta , \end{array}$$

where *δ* is a predetermined noninferiority margin corresponding to the difference in the rate of adverse outcomes between treatment and control. Whether or not the null hypothesis is rejected will depend largely on the choice of *δ*, which should be considered carefully. According to Weins, a good margin *δ* is “the smallest value that would represent a clinically meaningful difference or the largest value that would represent a clinically meaningless difference” [22]. It is therefore important to consider some confidence interval about *τ*_c_, the control proportion, in our case a range of proportions of adverse events at referral facilities. The smallest value *δ* that would represent a clinically meaningful difference should at least be outside of this control group confidence interval around *τ*_c_.

For clinical trials, for which a larger effect size often corresponds to higher drug efficacy, a common approach to selecting an appropriate and conservative *δ* uses “half the lower limit of the effect size 95% confidence interval for the active control therapy” [20] (p. 25). When evaluating adverse health outcomes, a lower treatment effect is preferred as it corresponds to lower rates of adverse outcome, and we therefore chose a similarly appropriate and conservative *δ* to equal half the 95% confidence interval for the difference between the control and treatment proportions. Consider an example where a hypothetical study cites a difference in rural and referral colonoscopy adverse event proportions of 0.3% ± 0.1%, using a 95% confidence interval. For a noninferiority test on the colonoscopy complication rate, we would select a *δ* = 0.05% as this value is half the margin of error. The *δ* is often assigned using data from historical studies and can present issues in clinical studies as the dependency between two separate noninferiority tests will be fairly high. This can be mitigated by increased sample size, as described in Tsong et al. [23], but as the methodology defined here concerns population‐level data, we consider this requirement to be satisfied.

### 4.3 Tests of noninferiority for logistic regression

Tests of noninferiority can be extended to the coefficients/adjusted odds ratios from a logistic regression analysis. We use logistic regression analysis to evaluate the relationship between the facility type and the odds of experiencing an adverse event, so that we can control for confounders such as the age or sex of the patient, socioeconomic status, the date or time of admission, and other factors that may bias our results. An appropriate noninferiority margin can still be chosen using the margin of error for the proportion of adverse outcomes of the control, and this margin can be applied to log‐odds ratios from either logistic regression or Firth penalized logistic regression models. Detailed instructions for the implementation of tests of noninferiority on adjusted odds ratios, including how to choose the noninferiority margin, are provided in Appendix A. The implementation differs between Firth penalized logistic regression and logistic regression analysis in that the former requires a likelihood ratio test on regression coefficients, while for the latter a Wald test can suffice.

### 4.4 Limitations

In any facility‐level comparison of health outcomes, differences in the coding of adverse events between facilities can bias the analysis. As inaccuracies in coding will inevitably affect the end results, we highlight the importance of spot‐checking volumes of interventions and their associated adverse events with hospital records when possible.

With a large‐scale evaluation spanning multiple hospitals, there could potentially exist differences between hospitals that would affect the adjusted odds of an adverse event. Hierarchical models can be used to better account for these differences, such as the use of a random intercept for each hospital. Evaluators should at least investigate the structure of the variance within facilities using intraclass correlation to assess the need for hierarchical modeling. As discussed, the consolidation of adverse events into overall indicators fails to account for differences in the severity of the various adverse events. Our mitigating suggestion was to present the individual proportions alongside the overall results, but this is not the most ideal option, as it is a raw comparison that does not account for confounding factors. Here we stress the need for an aggregate adverse‐outcome score that is modified by the severity of each adverse event. Derivation of such a tool would require extensive validation and clinician consultation but would lead to fairer and more granular facility‐level comparisons.

Finally, we stress that the methodology described should only apply to population‐level data, where the volume of adverse events is high enough to allow for adequate power when using logistic regression analysis.

## 5 CONCLUSION

The methodology presented here, applied to the evaluation of the Rural Surgical and Obstetrical Networks project, offers a clinically relevant approach to understanding quality in low‐volume rural services when compared to regional referral services through tests of noninferiority and penalized logistic regression models. This approach aligns with service delivery expectations and avoids unhelpful, and potentially destructive provider group comparisons. Tests of noninferiority, Firth penalized regression, and risk stratification were an effective approach for the study at hand and, we suggest, applicable to other rural health system quality projects.

## AUTHOR CONTRIBUTIONS

Gal Av‐Gay analyzed the data and prepared the first draft of the manuscript. All authors participated in the conception and design of the study and constructively revised the manuscript. Anshu Parajulee participated in data collection (ICD code mapping) and organization. Jude Kornelsen and Kathrin Stoll participated in and supervised the study throughout, and they share authorship. All authors commented on previous versions of the manuscript and approved the final version.

## CONFLICT OF INTEREST STATEMENT

The authors declare no conflict of interest.

## ETHICS STATEMENT

Ethics Approval: Rise UBC—H19‐01976.

## INFORMED CONSENT

The need for informed consent was waived for this study because no new patient data were reported.

## Supporting information

Supporting information.

## Data Availability

The data that support the findings of this study are available from Population Data BC. Restrictions apply to the availability of these data, which were used under license for this study. Data are available from Population Data BC with the permission of Population Data BC. Access to data provided by the Data Steward(s) is subject to approval but can be requested for research projects through the Data Steward(s) or their designated service providers. All inferences, opinions, and conclusions drawn in this publication are those of the author(s), and do not reflect the opinions or policies of the Data Steward(s).

## APPENDIX A TESTS OF NONINFERIORITY ON LOGISTIC REGRESSION COEFFICIENTS

### Tests of noninferiority for logistic regression

We use logistic regression analysis to evaluate the relationship between the treatment variable and the odds of experiencing an adverse event, where the treatment variable in our case is the type of facility (rural vs. referral). We can control for confounders such as the age of the patient, the socioeconomic status (income‐decile), the date or time of admission, and other factors that may bias our results. The coefficients in a logistic regression model correspond to adjusted log‐odds ratios, and tests on these coefficients can be done using a Wald confidence interval or likelihood ratio tests. We stress the importance of small sample considerations, especially when working with proportions that are very low (close to zero) or very high.

Say *p* is the probability that a patient experiences a complication, and *x*_1_ to *x*_*m*_ (where *m* is the total number of explanatory variables) are the explanatory variables, consisting of the treatment variable and other confounding continuous or discrete variables (patient comorbidity level, patient age, etc.). A logistic regression fits optimal coefficients *β*_0_ to *β*_*m*_ to the following model, where the left‐hand side describes the log‐odds log(*ω*) of experiencing a complication and the right describes a linear combination of explanatory variables.

(A1)
$$\begin{array}{l} \begin{array}{ccl} \log\left(\omega \right) & = & \log\left(\frac{p}{1-p}\right)=\beta_{0}+\beta_{1}x_{1}+\beta_{2}x_{2}\\ & & +\;\ldots \;+\beta_{m}x_{m}. \end{array} \end{array}$$

For binary explanatory variables taking on a value of 0 or 1, such as an indicator of rural versus referral facilities, *x*, where *x* = 1 represents rural and *x* = 0 referral, the coefficient itself can be viewed as a log‐odds ratio, such that the ratio of log‐odds of experiencing a complication changes by *β*, the fitted coefficient for variable *x*, between rural and referral facilities. In other words, the log‐odds of experiencing a complication are *β* times more or less at rural versus referral or comparator facilities/catchments.

The coefficient *β* can be expressed as an adjusted odds ratio *ψ* ^∗^, where $p_{t}^{*}$ and $\omega_{t}^{*}$ are the adjusted treatment (t) proportion and odds, respectively, $p_{c}^{*}$ and $\omega_{c}^{*}$ are the adjusted control (c) proportion and odds, respectively.

(A2)
$$\beta =\psi^{*}=\frac{\omega_{t}^{*}}{\omega_{c}^{*}}=\frac{p_{t}^{*}\left(1-p_{c}^{*}\right)}{p_{c}^{*}\left(1-p_{t}^{*}\right)}.$$

A “success” in this case corresponds to an adverse event and is considered undesirable; therefore, the hypothesis for testing that the treatment group is noninferior to the control group based on a prespecified threshold of *ψ*_0_ (*ψ*_0_ > 1) is as follows:

(A3)
$$H_{0}:\psi \geq \psi_{0},$$

$$H_{1}:\psi <\psi_{0}.$$

On the basis of the asymptotic normality of log(*ψ* ^∗^), the fitted coefficients have standard error $\hat{\sigma }$ ^2^ = SE^2^_*βj*_ (estimated using numerical methods) and a Wald test on the coefficients in a logistic regression model can be used to test the hypothesis of noninferiority as in expression (A3), for which the test statistic is

(A4)
$$\frac{\log\left(\psi^{*}\right)-\log(\psi_{0})}{\hat{\sigma }},$$

where the test statistic follows a standard normal distribution. The *p* value can be calculated as

(A5)
$$p\;\text{value}=P\left(Z<\frac{\log\left(\psi^{*}\right)-\log(\psi_{0})}{\hat{\sigma }}\right)=\Phi \left(\frac{\log\left(\psi^{*}\right)-\log(\psi_{0})}{\hat{\sigma }}\right),$$

where *Z* follows a standard normal distribution and the cumulative distribution function of the standard normal distribution is denoted by Φ(•). The null hypothesis will be rejected if the *p* value is < *α*.

In the context of a logistic regression model, choosing *ψ*_0_ by using half the margin of error for the difference between the active control treatment and the treatment can be done using expression (A2).

## References

[1] Iglesias S , Kornelsen J . An evidence‐based program for rural surgical and obstetrical networks. Rural Remote Health. 2018;18(4):4921. 10.22605/RRH4921 30507247

[2] Population Data BC . Services for researchers. Vancouver, BC: Population Data BC; 2022 [cited 2022 Oct 17]. Available from: http://www.popdata.bc.ca/index.php/researchers

[3] Canadian Institute for Health Information . Discharge abstract database metadata (DAD) [metadata]. Ottawa, ON: CIHI; 2022 [cited 2023 Aug 14]. Available from: http://www.cihi.ca/en/discharge-abstract-database-dad-metadata

[4] Perinatal Services BC . Perinatal data registry. Vancouver, BC: Perinatal Services BC; 2022 [cited 2022 Oct 17]. Available from: http://www.perinatalservicesbc.ca/our-services/data-surveillance/perinatal-data-registry

[5] Keeney S , Hasson F , McKenna H . Conducting the research using the Delphi technique. In: The Delphi Technique in Nursing and Health Research. Oxford: John Wiley & Sons, Inc; 2011. p. 69–83.

[6] Chimukangara M , Helm MC , Frelich MJ , Bosler ME , Rein LE , Szabo A , et al. A 5‐item frailty index based on NSQIP data correlates with outcomes following paraesophageal hernia repair. Surg Endosc. 2017;31(6):2509–2519. 10.1007/s00464-016-5253-7 27699515 PMC5378684

[7] Guller U , Hervey S , Purves H , Muhlbaier LH , Peterson ED , Eubanks S , et al. Laparoscopic versus open appendectomy: outcomes comparison based on a large administrative database. Ann Surg. 2004;239(1):43–52. 10.1097/01.sla.0000103071.35986.c1 14685099 PMC1356191

[8] Kassin MT , Owen RM , Perez SD , Leeds I , Cox JC , Schnier K , et al. Risk factors for 30‐day hospital readmission among general surgery patients. J Am Coll Surg. 2012;215(3):322–330. 10.1016/j.jamcollsurg.2012.05.024 22726893 PMC3423490

[9] Mansournia MA , Geroldinger A , Greenland S , Heinze G . Separation in logistic regression: causes, consequences, and control. Am J Epidemiol. 2018;187(4):864–870. 10.1093/aje/kwx299 29020135

[10] Muthén LK , Muthén BO . How to use a Monte Carlo study to decide on sample size and determine power. Struct Equ Model. 2002;9(4):599–620. 10.1207/s15328007sem0904_8

[11] Puhr R , Heinze G , Nold M , Lusa L , Geroldinger A . Firth's logistic regression with rare events: accurate effect estimates and predictions? Stat Med. 2017;36(14):2302–2317. 10.1002/sim.7273 28295456

[12] Girwar SAM , Jabroer R , Fiocco M , Sutch SP , Numans ME , Bruijnzeels MA . A systematic review of risk stratification tools internationally used in primary care settings. Health Sci Rep. 2021;4(3):e329. 10.1002/hsr2.329 34322601 PMC8299990

[13] Canadian Institute for Health Information . Introduction to CMG+ aggregation variables. In: Report. Ottawa, ON: CIHI; 2022 [cited 2022 Oct 17]. Available from: http://www.cihi.ca/en/access-data-and-reports/data-holdings

[14] Stoll K , Titoria R , Turner M , Jones A , Butska L . Perinatal outcomes of midwife‐led care, stratified by medical risk: a retrospective cohort study from British Columbia (2008–2018). Can Med Assoc J. 2023;195(8):E292–E299. 10.1503/cmaj.220453 36849178 PMC9970624

[15] Charlson ME , Pompei P , Ales KL , MacKenzie CR . A new method of classifying prognostic comorbidity in longitudinal studies: development and validation. J Chronic Dis. 1987;40(5):373–383. 10.1016/0021-9681(87)90171-8 3558716

[16] Althunian TA , de Boer A , Groenwold RHH , Klungel OH . Defining the noninferiority margin and analysing noninferiority: an overview. Br J Clin Pharmacol. 2017;83(8):1636–1642. 10.1111/bcp.13280 28252213 PMC5510081

[17] Canadian Institute for Health Information . Hospital harm indicator general methodology notes. Ottawa, ON: CIHI; 2021 [cited 2022 Oct 17]. Available from: http://www.cihi.ca/en/hospital-harm-project

[18] Savitch SL , Shah PC . Closing the gap between the laparoscopic and open approaches to abdominal wall hernia repair: a trend and outcomes analysis of the ACS‐NSQIP database. Surg Endosc. 2016;30(8):3267–3278. 10.1007/s00464-015-4650-7 26558910

[19] VanderWeele TJ , Ding P . Sensitivity analysis in observational research: introducing the E‐value. Ann Intern Med. 2017;167(4):268–274. 10.7326/M16-2607 28693043

[20] Rothmann MD , Wiens BL , Chan IS . Non‐inferiority trail considerationsIn: Design and analysis of non‐inferiority trials. 1st ed. New York: CRC Press; 2011. p. 21–25.

[21] Greene WL , Concato J , Feinstein AR . Claims of equivalence in medical research: are they supported by the evidence? Ann Intern Med. 2000;132(9):715–722. 10.7326/0003-4819-132-9-200005020-00006 10787365

[22] Wiens BL . Choosing an equivalence limit for noninferiority or equivalence studies. Controlled Clin Trials. 2002;23(1):2–14. 10.1016/s0197-2456(01)00196-9 11852160

[23] Tsong Y , Zhang J , Levenson M . Choice of δ noninferiority margin and dependency of the noninferiority trials. J Biopharm Stat. 2007;17(2):279–288. 10.1080/10543400601177384 17365224

