# Supplementary material for: Evaluating rural health outcomes: A methodological approach using population‐level data
Source: Health Care Sci. 2024 May 7;3(3):151–62. doi: 10.1002/hcs2.94 (PMC11212297; doi:10.1002/hcs2.94)
Supplement: Supplementary file 1 — Supporting information. [file HCS2-3-151-s001.pdf]

ICD_code	Complication_type	Complication
I26	General outcomes	Pulmonary embolism
I260	General outcomes	Pulmonary embolism
I269	General outcomes	Pulmonary embolism
N170	General outcomes	Acute renal failure
N171	General outcomes	Acute renal failure
N172	General outcomes	Acute renal failure
N178	General outcomes	Acute renal failure
N179	General outcomes	Acute renal failure
N990	General outcomes	Acute renal failure
A400	General outcomes	Sepsis
A401	General outcomes	Sepsis
A402	General outcomes	Sepsis
A4020	General outcomes	Sepsis
A4021	General outcomes	Sepsis
A403	General outcomes	Sepsis
A408	General outcomes	Sepsis
A409	General outcomes	Sepsis
A41	General outcomes	Sepsis
A410	General outcomes	Sepsis
A411	General outcomes	Sepsis
A412	General outcomes	Sepsis
A413	General outcomes	Sepsis
A414	General outcomes	Sepsis
A415	General outcomes	Sepsis
A4150	General outcomes	Sepsis
A4151	General outcomes	Sepsis
A4152	General outcomes	Sepsis
A4158	General outcomes	Sepsis
A418	General outcomes	Sepsis
A419	General outcomes	Sepsis
B377	General outcomes	Sepsis
R65	General outcomes	Sepsis
R650	General outcomes	Sepsis
R651	General outcomes	Sepsis
R652	General outcomes	Sepsis
R653	General outcomes	Sepsis
R659	General outcomes	Sepsis
T814	General outcomes	Surgical site infection
O860	Maternal outcomes, general	Surgical site infection
O86002	Maternal outcomes, general	Surgical site infection
O86004	Maternal outcomes, general	Surgical site infection
O86009	Maternal outcomes, general	Surgical site infection
Y60	General outcomes	Cut, puncture, perforation or haemorrhage
Y600	General outcomes	Cut, puncture, perforation or haemorrhage
Y601	General outcomes	Cut, puncture, perforation or haemorrhage
Y602	General outcomes	Cut, puncture, perforation or haemorrhage
Y603	General outcomes	Cut, puncture, perforation or haemorrhage
Y604	General outcomes	Cut, puncture, perforation or haemorrhage
Y605	General outcomes	Cut, puncture, perforation or haemorrhage
Y606	General outcomes	Cut, puncture, perforation or haemorrhage
Y607	General outcomes	Cut, puncture, perforation or haemorrhage
Y608	General outcomes	Cut, puncture, perforation or haemorrhage

Y609	General outcomes	Cut, puncture, perforation or haemorrhage
T812	General outcomes	Cut, puncture, perforation or haemorrhage
Y61	General outcomes	Foreign body
Y610	General outcomes	Foreign body
Y611	General outcomes	Foreign body
Y612	General outcomes	Foreign body
Y613	General outcomes	Foreign body
Y614	General outcomes	Foreign body
Y615	General outcomes	Foreign body
Y616	General outcomes	Foreign body
Y617	General outcomes	Foreign body
Y618	General outcomes	Foreign body
Y619	General outcomes	Foreign body
Y653	Anaesthesia outcomes	Failed tracheal intubation
T815	General outcomes	Foreign body
T8150	General outcomes	Foreign body
T8151	General outcomes	Foreign body
T8152	General outcomes	Foreign body
T8157	General outcomes	Foreign body
T8158	General outcomes	Foreign body
T8159	General outcomes	Foreign body
T816	General outcomes	Foreign body
T813	General outcomes	Wound disruption
S372	General outcomes	Bladder injury
S3720	General outcomes	Bladder injury
S37200	General outcomes	Bladder injury
S37201	General outcomes	Bladder injury
S3721	General outcomes	Bladder injury
S37210	General outcomes	Bladder injury
S37211	General outcomes	Bladder injury
S3729	General outcomes	Bladder injury
S37290	General outcomes	Bladder injury
S37291	General outcomes	Bladder injury
I21	General outcomes	Myocardial infarction
I210	General outcomes	Myocardial infarction
I211	General outcomes	Myocardial infarction
I212	General outcomes	Myocardial infarction
I213	General outcomes	Myocardial infarction
I214	General outcomes	Myocardial infarction
I219	General outcomes	Myocardial infarction
Z513	General outcomes	Blood transfusion
I60	General outcomes	Cerebrovascular accident (stroke)
I600	General outcomes	Cerebrovascular accident (stroke)
I601	General outcomes	Cerebrovascular accident (stroke)
I602	General outcomes	Cerebrovascular accident (stroke)
I603	General outcomes	Cerebrovascular accident (stroke)
I604	General outcomes	Cerebrovascular accident (stroke)
I605	General outcomes	Cerebrovascular accident (stroke)
I606	General outcomes	Cerebrovascular accident (stroke)
I607	General outcomes	Cerebrovascular accident (stroke)
I608	General outcomes	Cerebrovascular accident (stroke)
I609	General outcomes	Cerebrovascular accident (stroke)
I61	General outcomes	Cerebrovascular accident (stroke)

I610	General outcomes	Cerebrovascular accident (stroke)
I611	General outcomes	Cerebrovascular accident (stroke)
I612	General outcomes	Cerebrovascular accident (stroke)
I613	General outcomes	Cerebrovascular accident (stroke)
I614	General outcomes	Cerebrovascular accident (stroke)
I615	General outcomes	Cerebrovascular accident (stroke)
I616	General outcomes	Cerebrovascular accident (stroke)
I618	General outcomes	Cerebrovascular accident (stroke)
I619	General outcomes	Cerebrovascular accident (stroke)
I62	General outcomes	Cerebrovascular accident (stroke)
I620	General outcomes	Cerebrovascular accident (stroke)
I621	General outcomes	Cerebrovascular accident (stroke)
I629	General outcomes	Cerebrovascular accident (stroke)
I63	General outcomes	Cerebrovascular accident (stroke)
I630	General outcomes	Cerebrovascular accident (stroke)
I631	General outcomes	Cerebrovascular accident (stroke)
I632	General outcomes	Cerebrovascular accident (stroke)
I633	General outcomes	Cerebrovascular accident (stroke)
I634	General outcomes	Cerebrovascular accident (stroke)
I635	General outcomes	Cerebrovascular accident (stroke)
I636	General outcomes	Cerebrovascular accident (stroke)
I638	General outcomes	Cerebrovascular accident (stroke)
I639	General outcomes	Cerebrovascular accident (stroke)
I64	General outcomes	Cerebrovascular accident (stroke)
I80	General outcomes	Deep vein thrombosis
I800	General outcomes	Deep vein thrombosis
I801	General outcomes	Deep vein thrombosis
I802	General outcomes	Deep vein thrombosis
I803	General outcomes	Deep vein thrombosis
I808	General outcomes	Deep vein thrombosis
I809	General outcomes	Deep vein thrombosis
O871	Maternal outcomes, general	Deep vein thrombosis
O87102	Maternal outcomes, general	Deep vein thrombosis
O87104	Maternal outcomes, general	Deep vein thrombosis
O87109	Maternal outcomes, general	Deep vein thrombosis
O88	Maternal outcomes, general	Pulmonary embolism
O880	Maternal outcomes, general	Pulmonary embolism
O88001	Maternal outcomes, general	Pulmonary embolism
O88002	Maternal outcomes, general	Pulmonary embolism
O88003	Maternal outcomes, general	Pulmonary embolism
O88004	Maternal outcomes, general	Pulmonary embolism
O88009	Maternal outcomes, general	Pulmonary embolism
O881	Maternal outcomes, general	Pulmonary embolism
O88101	Maternal outcomes, general	Pulmonary embolism
O88102	Maternal outcomes, general	Pulmonary embolism
O88103	Maternal outcomes, general	Pulmonary embolism
O88104	Maternal outcomes, general	Pulmonary embolism
O88109	Maternal outcomes, general	Pulmonary embolism
O882	Maternal outcomes, general	Pulmonary embolism
O88201	Maternal outcomes, general	Pulmonary embolism
O88202	Maternal outcomes, general	Pulmonary embolism
O88203	Maternal outcomes, general	Pulmonary embolism
O88204	Maternal outcomes, general	Pulmonary embolism

O88209	Maternal outcomes, general	Pulmonary embolism
O883	Maternal outcomes, general	Pulmonary embolism
O88301	Maternal outcomes, general	Pulmonary embolism
O88302	Maternal outcomes, general	Pulmonary embolism
O88303	Maternal outcomes, general	Pulmonary embolism
O88304	Maternal outcomes, general	Pulmonary embolism
O88309	Maternal outcomes, general	Pulmonary embolism
O888	Maternal outcomes, general	Pulmonary embolism
O88801	Maternal outcomes, general	Pulmonary embolism
O88802	Maternal outcomes, general	Pulmonary embolism
O88803	Maternal outcomes, general	Pulmonary embolism
O88804	Maternal outcomes, general	Pulmonary embolism
O88809	Maternal outcomes, general	Pulmonary embolism
O904	Maternal outcomes, general	Acute renal failure
O90402	Maternal outcomes, general	Acute renal failure
O90404	Maternal outcomes, general	Acute renal failure
O90409	Maternal outcomes, general	Acute renal failure
O95	Maternal outcomes, general	Death
O95001	Maternal outcomes, general	Death
O95002	Maternal outcomes, general	Death
O95003	Maternal outcomes, general	Death
O95004	Maternal outcomes, general	Death
O95009	Maternal outcomes, general	Death
J690	Anaesthesia outcomes	Aspiration pneumonia
J954	Anaesthesia outcomes	Aspiration pneumonia
O290	Maternal outcomes, anaesthesia	Aspiration pneumonia
O29001	Maternal outcomes, anaesthesia	Aspiration pneumonia
O29003	Maternal outcomes, anaesthesia	Aspiration pneumonia
O29009	Maternal outcomes, anaesthesia	Aspiration pneumonia
O740	Maternal outcomes, anaesthesia	Aspiration pneumonia
O74001	Maternal outcomes, anaesthesia	Aspiration pneumonia
O74002	Maternal outcomes, anaesthesia	Aspiration pneumonia
O74003	Maternal outcomes, anaesthesia	Aspiration pneumonia
O74004	Maternal outcomes, anaesthesia	Aspiration pneumonia
O74009	Maternal outcomes, anaesthesia	Aspiration pneumonia
O890	Maternal outcomes, anaesthesia	Aspiration pneumonia
O89002	Maternal outcomes, anaesthesia	Aspiration pneumonia
O89004	Maternal outcomes, anaesthesia	Aspiration pneumonia
O89009	Maternal outcomes, anaesthesia	Aspiration pneumonia
T884	Anaesthesia outcomes	Failed tracheal intubation
O296	Maternal outcomes, anaesthesia	Failed tracheal intubation
O29601	Maternal outcomes, anaesthesia	Failed tracheal intubation
O29603	Maternal outcomes, anaesthesia	Failed tracheal intubation
O29609	Maternal outcomes, anaesthesia	Failed tracheal intubation
O747	Maternal outcomes, anaesthesia	Failed tracheal intubation
O74701	Maternal outcomes, anaesthesia	Failed tracheal intubation
O74702	Maternal outcomes, anaesthesia	Failed tracheal intubation
O74703	Maternal outcomes, anaesthesia	Failed tracheal intubation
O74704	Maternal outcomes, anaesthesia	Failed tracheal intubation
O74709	Maternal outcomes, anaesthesia	Failed tracheal intubation
O896	Maternal outcomes, anaesthesia	Failed tracheal intubation
O89602	Maternal outcomes, anaesthesia	Failed tracheal intubation
O89604	Maternal outcomes, anaesthesia	Failed tracheal intubation

O89609	Maternal outcomes, anaesthesia	Failed tracheal intubation
O742	Maternal outcomes, anaesthesia	Intraoperative cardiac dysrhythmia/arrest
O74201	Maternal outcomes, anaesthesia	Intraoperative cardiac dysrhythmia/arrest
O74202	Maternal outcomes, anaesthesia	Intraoperative cardiac dysrhythmia/arrest
O74203	Maternal outcomes, anaesthesia	Intraoperative cardiac dysrhythmia/arrest
O74204	Maternal outcomes, anaesthesia	Intraoperative cardiac dysrhythmia/arrest
O74209	Maternal outcomes, anaesthesia	Intraoperative cardiac dysrhythmia/arrest
O891	Maternal outcomes, anaesthesia	Intraoperative cardiac dysrhythmia/arrest
O89102	Maternal outcomes, anaesthesia	Intraoperative cardiac dysrhythmia/arrest
O89104	Maternal outcomes, anaesthesia	Intraoperative cardiac dysrhythmia/arrest
O89109	Maternal outcomes, anaesthesia	Intraoperative cardiac dysrhythmia/arrest
T410	Anaesthesia outcomes	Medication error, wrong dose
T411	Anaesthesia outcomes	Medication error, wrong dose
T412	Anaesthesia outcomes	Medication error, wrong dose
T413	Anaesthesia outcomes	Medication error, wrong dose
T414	Anaesthesia outcomes	Medication error, wrong dose
J952	Anaesthesia outcomes	Problems with airways in post-anaesthesia recovery, excluding aspiration pneumonia
J953	Anaesthesia outcomes	Problems with airways in post-anaesthesia recovery, excluding aspiration pneumonia
J955	Anaesthesia outcomes	Problems with airways in post-anaesthesia recovery, excluding aspiration pneumonia
J958	Anaesthesia outcomes	Problems with airways in post-anaesthesia recovery, excluding aspiration pneumonia
J9580	Anaesthesia outcomes	Problems with airways in post-anaesthesia recovery, excluding aspiration pneumonia
J9581	Anaesthesia outcomes	Problems with airways in post-anaesthesia recovery, excluding aspiration pneumonia
J9588	Anaesthesia outcomes	Problems with airways in post-anaesthesia recovery, excluding aspiration pneumonia
J959	Anaesthesia outcomes	Problems with airways in post-anaesthesia recovery, excluding aspiration pneumonia
S064	Maternal outcomes	Abscess/hematoma, among those with an epidural
G060	Maternal outcomes	Abscess/hematoma, among those with an epidural
G061	Maternal outcomes	Abscess/hematoma, among those with an epidural
G062	Maternal outcomes	Abscess/hematoma, among those with an epidural
O7021	Maternal outcomes	Major perineal tear
O70211	Maternal outcomes	Major perineal tear
O70214	Maternal outcomes	Major perineal tear
O70219	Maternal outcomes	Major perineal tear
O7022	Maternal outcomes	Major perineal tear
O70221	Maternal outcomes	Major perineal tear
O70224	Maternal outcomes	Major perineal tear
O70229	Maternal outcomes	Major perineal tear
O7023	Maternal outcomes	Major perineal tear
O70231	Maternal outcomes	Major perineal tear
O70234	Maternal outcomes	Major perineal tear
O70239	Maternal outcomes	Major perineal tear
O7028	Maternal outcomes	Major perineal tear

O70281	Maternal outcomes	Major perineal tear
O70284	Maternal outcomes	Major perineal tear
O70289	Maternal outcomes	Major perineal tear
O7029	Maternal outcomes	Major perineal tear
O70291	Maternal outcomes	Major perineal tear
O70294	Maternal outcomes	Major perineal tear
O70299	Maternal outcomes	Major perineal tear
O703	Maternal outcomes	Major perineal tear
O70301	Maternal outcomes	Major perineal tear
O70304	Maternal outcomes	Major perineal tear
O70309	Maternal outcomes	Major perineal tear
O72	Maternal outcomes	Postpartum hemorrhage, including with retained placenta
O720	Maternal outcomes	Postpartum hemorrhage, including with retained placenta
O72002	Maternal outcomes	Postpartum hemorrhage, including with retained placenta
O72004	Maternal outcomes	Postpartum hemorrhage, including with retained placenta
O72009	Maternal outcomes	Postpartum hemorrhage, including with retained placenta
O721	Maternal outcomes	Postpartum hemorrhage, including with retained placenta
O72102	Maternal outcomes	Postpartum hemorrhage, including with retained placenta
O72104	Maternal outcomes	Postpartum hemorrhage, including with retained placenta
O72109	Maternal outcomes	Postpartum hemorrhage, including with retained placenta
O722	Maternal outcomes	Postpartum hemorrhage, including with retained placenta
O72202	Maternal outcomes	Postpartum hemorrhage, including with retained placenta
O72204	Maternal outcomes	Postpartum hemorrhage, including with retained placenta
O72209	Maternal outcomes	Postpartum hemorrhage, including with retained placenta
O723	Maternal outcomes	Postpartum hemorrhage, including with retained placenta
O72302	Maternal outcomes	Postpartum hemorrhage, including with retained placenta
O72304	Maternal outcomes	Postpartum hemorrhage, including with retained placenta
O72309	Maternal outcomes	Postpartum hemorrhage, including with retained placenta
O902	Maternal outcomes	Postpartum hemorrhage, including with retained placenta
O90202	Maternal outcomes	Postpartum hemorrhage, including with retained placenta
O90204	Maternal outcomes	Postpartum hemorrhage, including with retained placenta
O90209	Maternal outcomes	Postpartum hemorrhage, including with retained placenta

O73	Maternal outcomes	Retained placenta, without hemorrhage
O730	Maternal outcomes	Retained placenta, without hemorrhage
O73002	Maternal outcomes	Retained placenta, without hemorrhage
O73004	Maternal outcomes	Retained placenta, without hemorrhage
O73009	Maternal outcomes	Retained placenta, without hemorrhage
O731	Maternal outcomes	Retained placenta, without hemorrhage
O73102	Maternal outcomes	Retained placenta, without hemorrhage
O73104	Maternal outcomes	Retained placenta, without hemorrhage
O73109	Maternal outcomes	Retained placenta, without hemorrhage
O7100	Maternal outcomes	Uterine rupture
O71001	Maternal outcomes	Uterine rupture
O71003	Maternal outcomes	Uterine rupture
O71009	Maternal outcomes	Uterine rupture
O7101	Maternal outcomes	Uterine rupture
O71011	Maternal outcomes	Uterine rupture
O71013	Maternal outcomes	Uterine rupture
O71019	Maternal outcomes	Uterine rupture
O7108	Maternal outcomes	Uterine rupture
O71081	Maternal outcomes	Uterine rupture
O71083	Maternal outcomes	Uterine rupture
O71089	Maternal outcomes	Uterine rupture
O7110	Maternal outcomes	Uterine rupture
O71101	Maternal outcomes	Uterine rupture
O71104	Maternal outcomes	Uterine rupture
O71109	Maternal outcomes	Uterine rupture
O7111	Maternal outcomes	Uterine rupture
O71111	Maternal outcomes	Uterine rupture
O71114	Maternal outcomes	Uterine rupture
O71119	Maternal outcomes	Uterine rupture
O7118	Maternal outcomes	Uterine rupture
O71181	Maternal outcomes	Uterine rupture
O71184	Maternal outcomes	Uterine rupture
O71189	Maternal outcomes	Uterine rupture
O715	C-section outcomes	Bladder, urethra injury
O71501	C-section outcomes	Bladder, urethra injury
O71504	C-section outcomes	Bladder, urethra injury
O71509	C-section outcomes	Bladder, urethra injury
K631	General outcomes	Bowel injury
S363	General outcomes	Bowel injury
S3630	General outcomes	Bowel injury
S36300	General outcomes	Bowel injury
S36301	General outcomes	Bowel injury
S3631	General outcomes	Bowel injury
S36310	General outcomes	Bowel injury
S36311	General outcomes	Bowel injury
S3639	General outcomes	Bowel injury
S36390	General outcomes	Bowel injury
S36391	General outcomes	Bowel injury
S364	General outcomes	Bowel injury
S3640	General outcomes	Bowel injury
S36400	General outcomes	Bowel injury
S36401	General outcomes	Bowel injury
S3641	General outcomes	Bowel injury

S36410	General outcomes	Bowel injury
S36411	General outcomes	Bowel injury
S3642	General outcomes	Bowel injury
S36420	General outcomes	Bowel injury
S36421	General outcomes	Bowel injury
S3645	General outcomes	Bowel injury
S36450	General outcomes	Bowel injury
S36451	General outcomes	Bowel injury
S3646	General outcomes	Bowel injury
S36460	General outcomes	Bowel injury
S36461	General outcomes	Bowel injury
S3649	General outcomes	Bowel injury
S36490	General outcomes	Bowel injury
S36491	General outcomes	Bowel injury
S365	General outcomes	Bowel injury
S3650	General outcomes	Bowel injury
S36500	General outcomes	Bowel injury
S36501	General outcomes	Bowel injury
S3651	General outcomes	Bowel injury
S36510	General outcomes	Bowel injury
S36511	General outcomes	Bowel injury
S3659	General outcomes	Bowel injury
S36590	General outcomes	Bowel injury
S36591	General outcomes	Bowel injury
S366	General outcomes	Bowel injury
S3660	General outcomes	Bowel injury
S36600	General outcomes	Bowel injury
S36601	General outcomes	Bowel injury
S3661	General outcomes	Bowel injury
S36610	General outcomes	Bowel injury
S36611	General outcomes	Bowel injury
S3669	General outcomes	Bowel injury
S36690	General outcomes	Bowel injury
S36691	General outcomes	Bowel injury
S367	General outcomes	Bowel injury
S3670	General outcomes	Bowel injury
S36700	General outcomes	Bowel injury
S36701	General outcomes	Bowel injury
S3671	General outcomes	Bowel injury
S36710	General outcomes	Bowel injury
S36711	General outcomes	Bowel injury
S3678	General outcomes	Bowel injury
S36780	General outcomes	Bowel injury
S36781	General outcomes	Bowel injury
S3679	General outcomes	Bowel injury
S36790	General outcomes	Bowel injury
S36791	General outcomes	Bowel injury
S369	General outcomes	Bowel injury
S3690	General outcomes	Bowel injury
S36900	General outcomes	Bowel injury
S36901	General outcomes	Bowel injury
S3691	General outcomes	Bowel injury
S36910	General outcomes	Bowel injury

S36911	General outcomes	Bowel injury
S3699	General outcomes	Bowel injury
S36990	General outcomes	Bowel injury
S36991	General outcomes	Bowel injury
O85	C-section outcomes	Peurperal sepsis, including endometritis
O85002	C-section outcomes	Peurperal sepsis, including endometritis
O85004	C-section outcomes	Peurperal sepsis, including endometritis
O85009	C-section outcomes	Peurperal sepsis, including endometritis
O900	C-section outcomes	Wound disruption
O90002	C-section outcomes	Wound disruption
O90004	C-section outcomes	Wound disruption
O90009	C-section outcomes	Wound disruption
O901	Maternal outcomes, general	Wound disruption
O90102	Maternal outcomes, general	Wound disruption
O90104	Maternal outcomes, general	Wound disruption
O90109	Maternal outcomes, general	Wound disruption
K912	Appendectomy outcomes	Gastrointestinal complication
K913	Appendectomy outcomes	Gastrointestinal complication
K918	Appendectomy outcomes	Gastrointestinal complication
K919	Appendectomy outcomes	Gastrointestinal complication
T810	Hernia repair outcomes	Hematoma and haemorrhage
K625	Colonoscopy	Heavy bleeding post-procedure
K922	Colonoscopy	Heavy bleeding post-procedure
T810	Colonoscopy	Heavy bleeding post-procedure
P10	Neonatal outcomes	Birth trauma
P100	Neonatal outcomes	Birth trauma
P101	Neonatal outcomes	Birth trauma
P102	Neonatal outcomes	Birth trauma
P103	Neonatal outcomes	Birth trauma
P104	Neonatal outcomes	Birth trauma
P108	Neonatal outcomes	Birth trauma
P109	Neonatal outcomes	Birth trauma
P11	Neonatal outcomes	Birth trauma
P110	Neonatal outcomes	Birth trauma
P111	Neonatal outcomes	Birth trauma
P112	Neonatal outcomes	Birth trauma
P113	Neonatal outcomes	Birth trauma
P114	Neonatal outcomes	Birth trauma
P115	Neonatal outcomes	Birth trauma
P119	Neonatal outcomes	Birth trauma
P12	Neonatal outcomes	Birth trauma
P120	Neonatal outcomes	Birth trauma
P121	Neonatal outcomes	Birth trauma
P122	Neonatal outcomes	Birth trauma
P123	Neonatal outcomes	Birth trauma
P124	Neonatal outcomes	Birth trauma
P128	Neonatal outcomes	Birth trauma
P129	Neonatal outcomes	Birth trauma
P13	Neonatal outcomes	Birth trauma
P130	Neonatal outcomes	Birth trauma
P1300	Neonatal outcomes	Birth trauma
P1301	Neonatal outcomes	Birth trauma
P1308	Neonatal outcomes	Birth trauma

P1309	Neonatal outcomes	Birth trauma
P131	Neonatal outcomes	Birth trauma
P132	Neonatal outcomes	Birth trauma
P133	Neonatal outcomes	Birth trauma
P1330	Neonatal outcomes	Birth trauma
P1338	Neonatal outcomes	Birth trauma
P134	Neonatal outcomes	Birth trauma
P138	Neonatal outcomes	Birth trauma
P139	Neonatal outcomes	Birth trauma
P14	Neonatal outcomes	Birth trauma
P140	Neonatal outcomes	Birth trauma
P141	Neonatal outcomes	Birth trauma
P142	Neonatal outcomes	Birth trauma
P143	Neonatal outcomes	Birth trauma
P148	Neonatal outcomes	Birth trauma
P149	Neonatal outcomes	Birth trauma
P15	Neonatal outcomes	Birth trauma
P150	Neonatal outcomes	Birth trauma
P151	Neonatal outcomes	Birth trauma
P152	Neonatal outcomes	Birth trauma
P153	Neonatal outcomes	Birth trauma
P154	Neonatal outcomes	Birth trauma
P155	Neonatal outcomes	Birth trauma
P156	Neonatal outcomes	Birth trauma
P158	Neonatal outcomes	Birth trauma
P159	Neonatal outcomes	Birth trauma
